# Energy, Poverty, and Health in Climate Change: A Comprehensive Review of an Emerging Literature

**DOI:** 10.3389/fpubh.2019.00357

**Published:** 2019-12-12

**Authors:** Sonal Jessel, Samantha Sawyer, Diana Hernández

**Affiliations:** ^1^Helibrunn Department of Population and Family Health, Columbia University Mailman School of Public Health, New York, NY, United States; ^2^Department of Sociomedical Sciences, Columbia University Mailman School of Public Health, New York, NY, United States

**Keywords:** energy insecurity, climate change, vulnerable populations, housing, health

## Abstract

Household energy is increasingly vital for maintaining good health. Unaffordable and inadequate household energy presents adverse consequences that are amplified by poverty and a changing climate. To date, the connections between energy, socioeconomic disadvantage, and well-being are generally underappreciated, and household energy connection with climate change is under-researched. Building on the energy insecurity framework, this review explores literature related to household energy, poverty, and health in order to highlight the disproportionate burdens borne by vulnerable populations in adequately meeting household energy needs. This paper is based on a comprehensive review of books, peer-reviewed articles, and reports published between 1990 and 2019, identified via databases including JSTOR and PubMed. A total of 406 publications were selected as having potential for full review, 203 received full review, and 162 were included in this paper on the basis of set inclusion criteria. From the literature review, we created an original heuristic model that describes energy insecurity as either acute or chronic, and we further explore the mediators and pathways that link energy insecurity to health. In the discussion, we posit that the extant literature does not sufficiently consider that vulnerable communities often experience energy insecurity bundled with other hardships. We also discuss energy, poverty, and health through the lens of climate change, making the criticism that most research on household energy does not consider climate change. This evidence is important for enhancing research in this field and developing programmatic and policy interventions as they pertain to energy access, affordability, and health, with special emphasis on vulnerable populations, climate change, and social inequality.

## Introduction

Global energy demand and consumption have increased substantially and are expected to continue rising (1); this is particularly evident in countries with strong economic growth such as China and India (2). The U.S. Energy Information Administration (EIA) has predicted that world energy use will increase by 28% by 2040 (2). In 2017 alone, global energy demand rose 2.1%—twice as much as in 2016 (3). As climate change worsens, the demand for more energy services and the strain on existing services will increase. As of 2017, residential energy consumption accounted for 20% of the total energy consumption across all sectors in the U.S. (4). Ensuring affordability, access, and adequate production of household energy is vital for maintaining individual and population-scale health and well-being. Household energy uses include cooking, lighting, heating, cooling, cleaning, and technological, medical, and other life-sustaining devices (5–7). Yet, millions of households around the world live without an adequate amount of energy (8).

Adequate access to energy is encumbered by limited and faulty infrastructure, affordability challenges, and service disruptions due to disasters and extreme weather events, often linked to climate change. This phenomenon, known as energy insecurity, is defined as the “inability to adequately meet household energy needs” (9). The energy insecurity framework includes physical, economic, and behavioral dimensions that lead to or exacerbate adverse health issues (9). The physical dimension includes poor housing quality, the structure of the home environment, and inefficient appliances (9). The economic dimension consists of affordability challenges (though it is not based merely on an economic ratio or threshold), while the behavioral dimension focuses on coping strategies, social vulnerabilities, and indicators of resilience (9). This conceptual framework helps us to understand the phenomenon of energy insecurity and its consequences. This framework parallels that of food insecurity—the “disruption of food intake or eating patterns because of lack of money and other resources” (10)—in that it reflects cost, quality, and health impacts.

In this article, we present the results of our review of the existing literature on energy insecurity and demonstrate the range of concerns and of approaches to resolving them. We propose the new terms- “acute” and “chronic” energy insecurity to further understand and break down household energy issues. Our findings suggest that the literature does not sufficiently consider the intersectionality of vulnerability types and multiple hardships. Furthermore, the use of numerous terms for household energy insecurity further compartmentalizes energy issues by geography and discipline, hampering the possibility for a comprehensive, or systematic literature base. This compartmentalization foregoes the opportunity to address energy insecurity as a complex, interdisciplinary, intersectional, and multidimensional issue, especially in the context of the pressing threat of climate change. For the sake of clarity, we provide below a brief overview of terms currently used to describe household energy issues as they relate to socioeconomic disadvantage, and in some cases, health.

## Household Energy Terminology

Many different terms have been used to describe the demand side of energy-related hardship. Researchers, policymakers, and practitioners have popularized terms such as fuel poverty, energy burden, energy vulnerability, and energy poverty, among others. These terms differ in their geographic usage and somewhat in their methods of measurement, but all similarly reference issues related to energy consumption and affordability. Energy burden and fuel poverty are mirroring terms that are used separately in different geographic regions; the former in the U.S. and the latter mostly in the United Kingdom (U.K.), Ireland, and New Zealand. Both terms are generally defined by an economic ratio whereby households that allocate more than 10% of their gross income for indoor energy expenses are considered energy burdened or fuel poor (9, 11–13). Energy poverty is generally attributed to the Global South and refers to the lack of modern energy services and low energy consumption (5, 14–20). Outcomes and indicators of energy poverty center on socioeconomic development, well-being, and poverty (21). A newer term, energy vulnerability, was developed to bridge the geographical research gap between fuel poverty and energy poverty in order to shed light on energy hardship as a global problem (14, 21). Importantly, these terms and their definitions have not yet incorporated the uncertain realities of climate change and its impact on energy, even though climate impacts are fundamentally rooted in how energy is produced and consumed, and its availability is threatened by the aftermath of extreme weather events, often caused by climate change.

Despite evidence of a strong association between energy access, affordability, and health, none of the above terms inherently focus on health. Research and policy tend to focus on the economic factors of energy burden, fuel poverty, and energy vulnerability, leading to financially-motivated interventions. For example, household-level financial subsidies (e.g., bill assistance) are a popular intervention, as opposed to more structural measures such as energy efficiency upgrades or adoption of clean energy technologies. Moreover, such an economic focus does not address the full scope of the problem, as it leaves out psychosocial and behavioral factors, among others, that contribute to energy hardship. Even energy poverty's focus on well-being and socioeconomic development omits housing quality and affordability constraints as a focus. By comparison, energy insecurity more broadly encompasses a wider spectrum of energy-related hardships that may be internal or external to an individual's experience of the phenomenon and is attuned to an expansive range of socioeconomic, psychological, and environmental determinants that produce energy-related hardship. The energy insecurity framework offers the opportunity to evaluate health predictors and outcomes in the context of climate change (22).

The present review provides an encompassing account of the relationship between energy, health, poverty, and climate change and the pathways by which these factors are interlinked. We address the critical gap in the importance of energy for population health (15, 23) by focusing not only on medical issues but also on the cycles of social disadvantage implicated in the nexus of energy, health, and poverty. We outline the impacts of global climate change on household energy access and how it contributes to the severity of health effects and discuss the need to accommodate growing demand on energy systems. This review provides evidence that health is a necessary consideration amidst increases in global energy demand. This is particularly important when: (1) developing methods for energy efficiency and production; (2) deciphering how to distribute and provide energy to low-income, marginalized, and vulnerable households equitably; and (3) preparing for climate change and acute threats to energy access.

## Paper Organization

We begin our analysis with a heuristic model linking the various factors that emerged during the review process, including the phenomena of chronic and acute energy insecurity (see **Figure 2**). This paper is organized into four thematic sections with subthemes. First, we propose new terminology to describe different manifestations of energy insecurity. Second, we review the evidence on energy insecurity and the social determinants of health by discussing the social patterning of energy insecurity by gender, age, health status, education, employment, socioeconomic status, and race. Next, we review evidence on the association between energy insecurity and place, noting the spatial inequalities in neighborhood resources and demographics that contribute to the increased likelihood that some community members will experience energy insecurity. Third, we outline findings regarding the connection between energy insecurity, housing quality, and home energy infrastructure by exploring the relationships between housing tenure, energy efficiency, and home age. Fourth, we highlight the salience of coping strategies and behaviors enacted by households experiencing energy insecurity and describe the health effects of temperature extremes, high-effort coping, and the depletion of resilience reserves. The *resilience reserve* is a framework that describes how resilience that should be preserved for use in a specific event, such as in response to a natural disaster, becomes depleted due to constant use in response to a greater prevalence of chronic daily struggles (24). To conclude, we summarize the findings of our review and describe the cumulative hardships of energy insecurity. In the discussion, we offer a critical analysis of the literature, highlighting that the research does not adequately consider the intersectionality of experiences of energy insecurity, infrequently employs an environmental justice framework, and lacks cohesive terminology. These critiques are discussed in relation to the growing wealth gap, increasing energy costs and demand, research into household energy in the Global South, and the inevitable impact of climate change.

## Methods

This review is based on a comprehensive search for relevant literature published between 1990 and 2018 and archived in JSTOR and PubMed. The literature review conceptually frames energy issues along the lines of climate change, health, and socioeconomic factors. The literature review search was conducted using a matrix of terms in varying combinations with one another. The search terms and their categories are outlined in Table 1. Terms from each category were searched along with terms from other categories. For example: (1) [(energy insecurity) AND (poverty) AND (climate change) AND (health)]; and then, (2) [(fuel poverty) AND (poverty) AND (climate change) AND (health)]. A term from the “Social,” “Health,” and “Energy” category was used in every search because one of our inclusion criteria is that articles discuss the relationship between household energy, health, and socioeconomic status. We did not include “climate change” as a required term for every search, as part of the review is analyzing the extent to which the existing literature on household energy considers climate change. We excluded sources about energy and climate change that did not have a health outcome or a socioeconomic focus. Any source that did not discuss energy at the household, neighborhood, or community level was also excluded. Beyond this, our criteria were purposefully broad in order to capture the breadth of topics related to household energy use, health, and climate.

**Table 1:** Search terms used in the literature review.

**ENERGY TERMS**
• *Fuel poverty*
• *Energy poverty*
• *Energy burden*
• *Energy vulnerability*
• *Energy insecurity*
• *Energy inefficiency*
**HEALTH TERMS**
• *Health*
• *Chronic illness or conditions*
• *Excess winter mortality, excess heat death*
**HOUSEHOLD TERMS**
• *Household, Housing, or Home*
• *Neighborhood or Community*
**SOCIAL TERMS**
• *Low-income*
• *Poverty*
• *Socioeconomic Status*
• *Vulnerable population*
• *Social vulnerability*
• *Resilience*
• *Coping*
• *Housing/Food insecurity*
**CLIMATE TERMS**
• *Natural disaster*
• *Extreme Weather event*
• *Climate change*
• *Global warming*
• *Heat wave*
• *Hurricane*
• *Power outage or shutoff*

**Figure 1:**
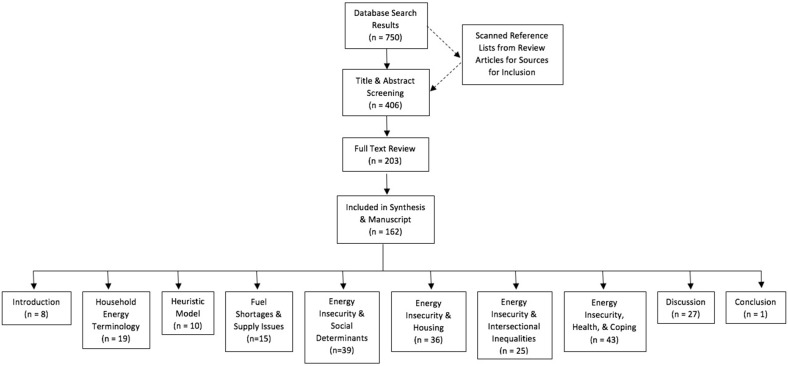
Flow chart of literature selection and review process.

The search was limited to articles in English. Books, peer-reviewed articles, and reports were included, and all other source types were excluded. Our initial database search plus additional articles added from reviewing the reference sections of various sources yielded 750 results. After discarding duplicate sources and literature that was not relevant based on title, we had 406 sources. These sources underwent title and abstract screening. At this point, we identified 203 sources for full review. Of these, 162 were analyzed and incorporated into the final manuscript (Figure 1). Some sources out of the 203 identified were not included in the final manuscript due to the subsequent irrelevance of their topic once the topics discussed in the manuscript had been refined during editing.

## Heuristic Model

**Figure 2:**
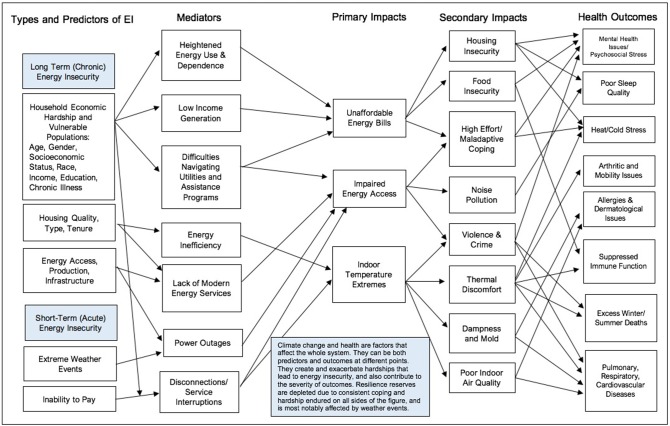
Connection between household energy insecurity (EI) and health.

Energy insecurity can impact health in a multitude of ways. Studies on energy insecurity encompass not only direct but also indirect health impacts because they consider social determinants of health and the coping strategies people use in response to energy insecurity. Inadequate household energy has been linked to the following health outcomes for both adults and children: cardiovascular, pulmonary, and respiratory illnesses; cancer; arthritis; acute hospitalization; excess mortality in summer and winter; and anxiety, depression, and stress (9, 13, 22, 25). Indirect health impacts, such as food insecurity, are also associated with energy insecurity (6, 26–30). It is important to highlight how indirect health problems contribute to and compound direct health impacts related to household energy insecurity. With health as an endpoint, our innovative, original heuristic model (Figure 2) tracks the multifaceted pathways that directly and indirectly link energy insecurity to health by distinguishing between chronic and acute energy insecurity.

## Defining Chronic and Acute Forms of Energy Insecurity

Energy insecurity is a complicated, multifaceted issue that may be best understood by parsing out its various forms—acute and chronic energy insecurity. For this reason, we propose the incorporation of these terms into the lexicon of energy insecurity work. *Chronic energy insecurity* is a long-term issue that can arise from a consistent inability to afford or access adequate energy to meet household needs. An example of chronic energy insecurity would be living in a home that is consistently cold because the cost of heating is unaffordable. The lack of adequate household energy is often predicated on a number of social and demographic factors including age, gender, socioeconomic status, education, income, race, and employment status. These characteristics may have implications for earnings potential, can determine a household's ability to navigate available resources, and affect access to efficient housing and energy use patterns.

*Acute energy insecurity* is a short-term issue that tends to arise from infrastructural, maintenance, environmental, or other external sources that disrupt access to energy sources. Some examples of acute energy insecurity include a power outage from a hurricane or a gas shutoff from a reported leak. Interestingly, chronic energy insecurity can lead to a significant crisis point of acute energy insecurity, such as shut-offs due to non-payment. Shutoffs are an acute form of energy insecurity because, for the most part, they are short-lived, and services are reinstated upon cost recovery by the energy service provider. Three primary forms of acute energy insecurity—fuel shortages and supply issues, power outages, and shut-offs—and the links between service interruptions and ill health are described next.

## Fuel Shortages and Supply Issues

Health impacts from energy access issues (when infrastructure is available) can arise when demand exceeds supply via two primary routes: (1) fuel shortages and (2) capacity of the energy infrastructure to handle excess demand during extreme weather. Fuel shortages across the world also indirectly impact health by increasing fuel cost and making sources unaffordable, leading to inaccessibility. Sometimes, other resources necessary for life are contingent on energy access. For example, during a fuel shortage, rural Iñupiaq Eskimo villages in Alaska's Northwest Arctic Borough were unable to access clean water because they did not have heat or electricity to prevent pipes from freezing (31). The shortage led to increased rates of infectious disease, hygiene-related diseases, and pneumonia (31). Fuel shortages may become worse as climate change worsens, increasing the prevalence of health issues related to energy insecurity (32).

In other instances, the energy infrastructure has proven incapable of tolerating higher than normal demands. A polar vortex, which occurred in January 2019 across the United States, brought extremely cold temperatures that strained energy systems. Some parts of the Midwest reached temperatures as low as minus 38 degrees Fahrenheit (33). An estimated 21 people died across the country from causes directly related to the polar vortex, such as death by hypothermia while indoors (34). One woman in Milwaukee died from the cold when her thermostat malfunctioned (34). In many cities, gas companies ordered households and industry to lower their heat in order to prevent citywide gas shortages (33). This compromised capacity is related to reliance on an aging infrastructure and increased demand, which is likely to be an issue over time and as weather patterns become more extreme as a result of climate change.

### Power Outages

Infrastructural issues, extreme weather, and natural disasters may result in power outages and energy service interruptions. Short-term power outages often occur for the following reasons: (1) older and less reliable infrastructure; (2) overloading from high electricity demand; (3) household maintenance defects; and/or, (4) other systems fail (35). Long-term power outages are typically a result of disasters such as hurricanes. In 2017, Hurricane Maria left Puerto Rico's residents without power for an unprecedented average of 84 days. An estimated 83% of these households were in the most remote regions of Puerto Rico (36). These outages interrupted healthcare for people with chronic illnesses who required care and medication (36). Moreover, the death toll attributable to Hurricane Maria in Puerto Rico was estimated to be 4,645 people, and 9.5% of those deaths were due to an inability to access electricity needed for respiratory equipment (36). The adverse impacts of service interruptions—both short- and long-term—are ubiquitous. Elevator shutdowns impede movement, transit systems close, medical device access can be cut off, blackouts can increase crime and cause accidents, food can spoil in warming refrigerators, temperature control can be lost, and much more (35, 37–39).

### Shutoffs

Affordability challenges may lead to a different type of service interruption—shutoffs or disconnections due to non-payment. It is challenging to measure a national prevalence rate of shutoffs, because utility companies seldom, if ever, report which and how many households are shut off, and these data are generally not publicly available. Hernández and Laird (40) used the Residential Energy Consumption Survey (RECS), administered by the Energy Information Administration (EIA), to analyze the prevalence of disconnection notices and service disconnections based on a nationally representative sample of US households. Hernández and Laird (40), presenting their results at the annual conferences of the American Sociological Association (ASA) and the American Public Health Association (APHA), demonstrated that in 2015 an estimated 3% of US households were disconnected and another 15% received a disconnection notice, suggesting that there is indeed some hardship. Consistent with previously observed patterns of inequality, the authors also found that low-income households, African-American and Latino households, households with children, renters, and people living in older and poorly insulated homes were most likely to receive disconnection notices and service interruptions. Hernández and Laird's (40) research further explores the coping strategies that families resort to, such as forgoing food and medicine and keeping homes at an unhealthy temperature, that compromise health and may even lead to death. Hernández and Laird (40) found that households rely on these strategies to prevent and respond to the threat and occurrence of shutoffs (40).

### Service Interruptions and Health

Shutoffs and power outages have a number of direct health impacts (30, 35, 37). Temperature-related issues such as hypothermia and heat stress increase when interruptions occur at times of extreme outdoor temperature. Chronic health conditions are strained by short- and long-term service interruptions in electricity and hot water services (30, 41). Additionally, people with chronic illnesses, particularly those with cardiovascular, respiratory, and renal diseases, are often forced to seek outside medical care during interruptions, increasing the rate of hospitalization (35, 42). Rates of all-cause and external-cause mortality are significantly higher during power-outage periods, especially when outages are due to weather events such as hurricanes, heatwaves, and snowstorms (30, 35). Households that have faced threats of shutoffs have reported more long-term mental health issues stemming from financial, physical, and environmental stress, as well as fears and anxiety over potential future service interruptions (13, 43).

## Energy Insecurity and Social Determinants

Both acute and chronic energy insecurity are influenced by the social determinants of health, which are defined by the Centers for Disease Control and Prevention (CDC) as the political, economic, and social circumstances in which people live, work, and play (44). These determinants include factors such as gender, age, health status, education, employment, socioeconomic status, and race. Here, we review literature indicating that the social determinants of health play an important role in predicting the existence of household energy difficulties and outline the importance of making a connection between the impact of climate change on social determinants of health and energy insecurity. As climate change increases the frequency, duration, and magnitude of extreme weather events, it is important to consider the populations that are most vulnerable to the impacts of such events within the context of household energy use (45, 46).

### Age and Life Course Vulnerability

Age-related vulnerabilities (i.e., being young or elderly) and exposure to environmental hazards are predictors of energy insecurity. The elderly, because they are at a higher risk of experiencing medical events during heatwaves than other populations due to a combination of social isolation, heightened physiological vulnerability, and the likelihood of not having air conditioning, are more likely to endure heat stress (46–48). Children are at risk of asthma, especially those living in urban, low-income communities, and are therefore in more need of adequate energy for ventilation and temperature control (49–51). Both the elderly and children spend more time in their home environments than other groups (1), increasing their exposure to the health risks of energy insecurity (52).

### Education: Literacy and Impacts on Academic Achievement

Education, as it relates to energy insecurity, also has implications for health. Lacking sufficient knowledge and ability to navigate the bureaucracy of utility companies makes it difficult for less-educated households to address and prevent energy insecurity. Knowing how to access resources such as financial subsidies or medical certifications to prevent shutoffs requires knowledge of how such bureaucratic systems work. Understanding the risks associated with using alternative methods to address energy needs also requires education. On average, those with less educational attainment have more limited income potential, making it more difficult to afford and make energy payments (6, 15).

Energy insecurity also perpetuates a cycle of lower educational attainment, most notably for children. Environmental stress and financial insecurity can lead to mental health issues and result in worse educational outcomes (53). The stress of energy hardship is associated with behavioral problems in children, whereby they are more likely to have low academic motivation, difficulty concentrating, and often act out (54, 55). Children experiencing energy insecurity and food insecurity (discussed in detail in subsequent sections) are also more likely to experience intensified behavioral issues such as depression, rule-breaking behavior, and somatic complaints (54). Asthmatic children in energy-insecure households with poor air quality miss more days of school due to illness than do non-asthmatic children (55, 56). Homes that use unsuitable energy sources expose children to toxic gasses that impair cognitive development. Additionally, children living in energy-insecure households often have trouble focusing on their homework due to noise pollution from generators, other loud energy sources, and open windows, which can lead to lower academic success (57). As a coping strategy, some households confine energy use to specific rooms to keep energy bills low or because there are limitations in their heating and cooling systems; this forces all residents to be in the same room, resulting in homework distractions and crowding, among other issues. Poor or restricted lighting to certain rooms also makes schoolwork and reading for pleasure more challenging.

### Employment

Energy insecurity perpetuates the detrimental health effects associated with low-income employment. Many low-paying jobs require work in extreme temperature conditions (58), such as farming in excess heat or working in refrigerated warehouses. Those working in extreme temperature conditions are also more likely to be experiencing energy insecurity at home because they are low-income. Exposure to thermal discomfort at work and at home has a cumulative effect on temperature-sensitive health problems. Members of low-income households are also more likely to work multiple jobs to pay bills and support their families, meaning they are often physically absent from the home. This absence of caretakers can affect children's developmental needs and contribute to social deprivation and caretaker stress. Single mothers are more likely to be primary caretakers and often experience augmented impacts because they do not have a partner with whom they can share the burden and responsibility. Single mothers are also more at risk of experiencing energy insecurity compared to other groups (9, 23).

### Socioeconomic Status and Household Income

Socioeconomic factors are a predictor of energy insecurity. In most households, utilities make up a substantial portion of living costs, and in low-income households, this proportion is much greater (9, 30, 59). Therefore, it can be difficult for low-income households to pay for enough energy to meet household needs and also afford other expenses (9, 30). Households at or near the Federal Poverty Level (FPL) are significantly more burdened by energy insecurity than other socioeconomic groups (9). A brief look at 2011 American Community Survey (ACS) data on the characteristics of people experiencing the gap between energy affordability and unaffordability found that 44% of low-income families (defined as below 200% of the FPL) experience economic energy insecurity compared to 2% of families who are not low-income (60). A 2016 report by the American Council for an Energy-Efficient Economy (ACEEE) found that lower-income households experience higher median energy burden (7.2%), defined as the percentage of household income spent on energy bills, whereas non-low-income households experienced a median energy burden of 1.5% (61). Furthermore, a recent study found that low-income households in the studied U.S. cities spend on average from 10 to 20% of their income on energy bills, while wealthier households spend on average between 1.5 to 3 percent on energy bills despite being the higher consumers (59). The 2015 RECS found that the lower the income, the higher the energy expenditure and energy consumption per square foot of the home due to a number of potential factors (62). For example, socioeconomic status has major implications for affordability, access, and levels of environmental hazards in the home. Additionally, low-income households are often unable to afford utility bills and therefore live without adequate energy due to heightened conservation and/or an inability to upgrade energy-related appliances and systems (59).

### Race

Race is another social determinant of health that can predicate energy insecurity. Minorities tend to suffer from higher rates of energy cost burden than non-Hispanic whites in United States' cities (59). In the U.S., African Americans suffer more from energy insecurity than do any other racial groups (23, 60, 63). Of surveyed households with an African-American head of household (HOH) and children under the age of 18, 35% reported facing energy insecurity compared to 21% of Latino HOHs with children under 18 and 14% of Caucasian HOHs with children under 18 (60). Across all income levels, Black families still maintained the highest rates of energy insecurity (60). In the Washington Heights neighborhood in New York City, energy-insecure households were more likely to be black and Hispanic/Latino, low-income, and have less education (64). In Detroit, a study found that African-American households were twice as likely to be behind on utility payments and three times more likely to suffer from arrearage or shut-offs than white households (65).

When considering racial disparities, the association between environmental hazard exposure and geographical location is stronger for Black and Latino communities than for other racial groups (66). The health impacts of energy insecurity are compounded for racial-minority households that live in areas with high rates of exposure to environmental hazards energy inefficiencies (59, 66). The disparity in the impacts of acute energy insecurity is especially apparent for minority racial groups. For example, a study by O'Neill et al. (67) found that during heatwaves in four different cities across the U.S., the rate of air conditioning use was more than two times higher among the white population than the black population, suggesting that black residents did not have access to or could not afford to use air conditioning at the same rate as white residents. Additionally, the mortality rate of black residents was significantly higher than that of other racial groups across all four cities (67). This trend could be attributed to low income levels and unaffordable electricity, which disproportionately impact racial and ethnic minority households.

### Gender

Gender is another social factor associated with energy insecurity that has direct and indirect health implications. In the Global South, women and girls, who are responsible for cooking, use biomass fuel, resulting in high rates of respiratory illness. Respiratory illness from solid-fuel cooking is one of the greatest causes of premature mortality globally (5, 7, 15, 68). In the Global North, women are more likely to be caretakers and spend more time at home, increasing their rate of exposure to other energy inefficiencies. Single mothers are especially vulnerable because they take on financial and psychosocial burdens alongside the responsibility of being the sole caretaker (23). It is important to note, however, that the literature in this review often fails to recognize that the separation of how gender relates to energy insecurity in the Global North vs. the Global South is reductive and essentialized, as all of the mechanisms by which women and girls are impacted by energy issues crosscut both spheres of the world.

### Health Status

Illness and chronic health problems often determine energy demand and have implications for energy consumption, thereby making poor health both a predictor and outcome of energy insecurity. Residents with health conditions such as cardiovascular, pulmonary, and respiratory diseases and arthritis are sensitive to temperature extremes, meaning that a home that is too cold or too hot can exacerbate and worsen symptoms (13, 69–71). People living with a chronic health condition may be especially reliant on energy-dependent devices for treatment or maintenance of their condition, lowering their ability to withstand inadequate or unavailable energy services. For example, patients with kidney disease, chronic obstructive pulmonary disease (COPD), and cardiovascular disease (CVD) rely on dialysis and oxygen machines that require electricity (72) and diabetics must refrigerate their insulin. Cancer patients in active treatment need more heat (73), and those suffering from hypertension are more susceptible to cold stress (74). Not only do people with chronic health conditions have an increased need for energy, but they also spend more time in their households, further increasing energy use (75).

## Energy Insecurity and Housing

A number of household characteristics beyond location predict the existence of unmet household energy needs. The type of housing, whether owned or rented, its level of energy efficiency, and its age are all associated with energy insecurity. Low-income and minority residents face a higher proportion of difficulties related to these housing characteristics.

### Housing Tenure and Type

Energy insecurity is affected by housing tenure because renting and owning can lead to unique challenges that perpetuate energy insecurity. Low-income renters often face difficulties affording household costs (76) and tend to spend the greatest portion of their income on energy bills when compared to other socioeconomic groups (77). They also tend to live in the most structurally deficient homes due to a lack of weatherization and efficiency upgrades (77). Energy insecurity can become a chronic issue, partly because low-income renters have limited ability to persuade landlords to maintain proper upkeep and implement effective modifications related to energy efficiency (43, 78). They also have limited social and economic capital to afford self-repairs or to hold landlords accountable through the court system (43). Low-income owners are also at risk for energy insecurity due to the high cost of upgrading homes to higher efficiency standards or because buying a home that is already efficient is expensive. Homeowners are often responsible for the entire burden of utility bills and other operational costs, including property taxes, home insurance, and water, garbage, and sewer costs. The stress of low-income housing on both renters and owners is associated with adverse mental health outcomes and poor self-rated health (43, 76).

Housing type also influences rates of energy insecurity. Similar to homeowners, renters of single-family units face difficulties because they are responsible for the entire cost burden. Multifamily housing can be more advantageous than single-family housing because there is a shared cost with property owners. However, residents in multifamily housing and low-income tenants often lack control over the conditions (e.g., heat) of their units and have restricted ability to combat energy insecurity, mostly due to financial constraints and lack of control over housing infrastructure (9). The New York City Housing Authority (NYCHA), the country's largest public housing authority, has faced notorious housing quality and energy infrastructure issues that have plagued residents by severely compromising their housing experience and, likely, their physical, and mental health. One challenge is that NYCHA is not subject to the same maintenance regulations as private housing or developers, resulting in structural deficiencies and energy inefficient housing, attributable in part to deferred maintenance (79). Public housing is not alone in this regard. Rental housing, including subsidized, or affordable housing, presents challenges for renters since the property owners determine the level of energy efficiency and other aspects of housing quality. In most cases, there are no guidelines that stipulate a minimum level of efficiency, particularly in older housing that was constructed when building codes were less focused on sustainability. This conundrum, known as the “split incentive,” occurs when the incentive structure for an asset is not equally beneficial to both parties. In such cases, the deciding actor works in their own best interest, as is the case with owners who dictate the terms of housing without consideration of tenancy. Previous research demonstrates that subsidized housing recipients face an increased burden because they are more likely to rent from private landlords who neither weatherize nor optimize energy efficiency due to upfront costs and administrative encumbrances, which generally privileges the property owners and negatively impacts the tenants both economically and experientially (77, 80).

Beyond rental and multiunit housing, people living in manufactured housing, such as trailers or mobile homes, are disproportionately impacted by physical energy insecurity. These housing structures are often not well-insulated or weatherized, so residents tend to spend a high proportion of their income on energy and heating bills. For example, the ACEEE found that mobile home residents are more likely to be energy burdened (61). Residents living in these manufactured housing types also tend to be low-income and therefore the least able to sustain high utility bill costs or afford general maintenance (81).

### Energy Inefficiency

Energy inefficiency is a common housing problem and aspect of energy insecurity that has serious health implications. Energy inefficiency is marked by poor insulation, drafts, leaks, and other points of intrusion of the outside elements that make it difficult to control indoor temperatures (82–84). Other structural deficiencies and poor housing quality conditions, such as a lack of central air conditioning and proper ventilation, can also lead to high utility bills and unsafe conditions (85). Energy inefficiency caused by poor housing quality and structural deficiencies spurs costly utility bills that are unaffordable for low-income people (86) Poor energy efficiency has been associated with an increase in household dampness (85), which is associated with worsened arthritis symptoms, dizziness, headaches, and fevers (79), and increases the presence of mold, exacerbating medical conditions such as allergies, eczema, and asthma (69, 87–90). Energy inefficiency is also associated with an increase in a number of thermal-related illnesses (85), and homes with poor ventilation and outside air infiltration have more dust mites and cockroach feces, which are known to exacerbate or lead to acute respiratory illnesses (27, 91–93). Households that are unable to open windows (see also the section entitled Heat Stress and Forbearance) have the additional risk of dampness as a result of obstructed airflow (57).

A popular intervention for older and/or poorly constructed homes is retrofitting (87). However, energy efficiency without attention to ventilation can lead to excess tightness in the building envelope, thereby obstructing airflow and exacerbating the aforementioned health issues related to ventilation and air quality (94). Air-tightness due to energy efficiency improvements is also associated with increased levels of radon, which significantly increases the risk of lung cancer (95, 96).

### Age of Housing

Older housing is a frequent contributor to energy insecurity because much of the aged housing stock around the world is neither weatherized nor energy-efficient, which results in an increased prevalence of thermally inadequate home environments (97). Low-income, older and minority householders are often relegated to substandard living conditions, in part due to residence in older housing that has not been renovated (98). The effect of older, less efficient housing on energy insecurity has been studied mostly in the fuel poverty literature from northern Europe and New Zealand, where the regularity of colder outdoor temperatures heightens the need for consistent indoor heating (82, 99). Excess winter deaths are a measure of mortality as a result of cold homes, a problem known to be caused by a lack of insulation (13, 47, 69, 84, 99–101). Heat stress is a common health effect of hotter outdoor temperatures, whereas newer technology such as centralized air conditioning may not exist in older homes. A lack of air conditioning contributes to heat stress, excess deaths, and hospitalizations during heatwaves (67, 102). Therefore, the use of newer technology is important for health and safety, particularly as it relates to the prevention of premature death.

Newer homes are subject to current housing codes, many of which include public health considerations, and tend to be more energy-efficient and have fewer maintenance issues (97). Many states have ventilation standards, for example, which can combat mold from dampness and therefore reduce asthma symptoms (98). Regulations on toxin levels in homes, such as through initiatives for lead-free homes, also exist. It is, of course, easier to control risks that are never introduced into the housing sphere. Therefore, living in new housing stands to benefit occupants. However, the most vulnerable groups are often the least likely to benefit from such advantages.

## Energy Insecurity and Intersectional Inequalities

Research has demonstrated that socioeconomic status and race are predictors of neighborhood, place, and presence of other hardships that can lead to or exacerbate energy insecurity. For example, residents in low-income and minority neighborhoods tend to experience issues such as increased exposure to environmental hazards, a lack of investment in housing maintenance, and poorer quality housing, all of which contribute to energy insecurity. Moreover, poverty and material hardship are complex issues in and of themselves, whereby the inability to meet basic needs extends far beyond any one category. In this section, we explore the overlapping issues that intersect with energy insecurity.

### Socioeconomic Status, Race, and Place

The coalescence of socioeconomic status, race and neighborhood factors can lead to or exacerbate energy insecurity and present other hardships as well. For example, racial residential segregation, a proxy for concentrated neighborhood disadvantage, is a demonstrated predictor of energy insecurity (103). Black and Latino/a-headed households are more likely to live in an energy-insecure household because of their home's lack of energy efficiency (103). For example, NYCHA housing, predominantly inhabited by minorities, is facing a backlash over its dilapidation and lack of maintenance, which has resulted in widespread power outages, lack of heating, and the presence of mold and lead problems (79). Of these issues, NYCHA has been criticized severely for the persistent lack of available heat in its properties due to faulty boilers (104). In the winter of 2017/2018, ~80% of NYCHA residents faced a heat outage, which lasted 48 hours on average (105). Some advocates state that the difference in regulations for NYCHA housing compared to private housing is unjust and disproportionately disadvantages low-income communities of color (79, 106).

Low-income and minority neighborhoods collectively bear the brunt of more environmental hazards in and outside of their individual households (50, 107). For example, the siting of highly-polluting sources such as bus depots, landfills, highways, and plants or factories tend to be located in low-income communities. Low-income homes are also more likely to have maintenance defects, rodents, mold, and other poor housing conditions (66). Households in low-income and minority neighborhoods are also more likely to be overcrowded; this is especially true for immigrant populations in New York City and other urban areas. Overcrowded homes are associated with psychosocial stress, disease outbreaks, and higher asthma rates; they are also a predictor of the existence of other physical and social housing-related hardships that contribute to the burden of disease (93, 98, 108).

Furthermore, institutional and systemic racism and place-related social factors are drivers of higher rates of energy insecurity for minority populations. Ethnic minorities, immigrants, and indigenous groups are some examples of people who experience housing discrimination (57), a barrier to accessing more energy-efficient homes. Gentrification in many urban areas in the U.S is another social process that perpetuates racial and ethnic disparities in energy insecurity prevalence. Gentrifying or newer residents are less likely to experience energy insecurity or have an energy inefficient home compared to longer-term residents who live in older households in the area (97). Long-term residents of Washington Heights in New York City, for example, are Dominican immigrants and African Americans, and they suffer far more energy insecurity than new neighborhood residents (64).

### Neighborhoods and Spatial Inequality

Energy insecurity, as we have noted, is highly correlated with spatial inequality, where residents of different neighborhoods are more or less likely to experience energy insecurity due to their neighborhood's economic, environmental, and social makeup. The mean annual energy use intensity (EUI), which is a proxy for energy insecurity by way of high energy use from low housing efficiency, is much higher in urban areas that have lower socioeconomic status, less education, and more racial minority dwellers (65, 103). In cities that are more racially segregated, neighborhoods with low-income and minority populations are more likely to suffer from difficulties in affording or accessing energy (65, 109). As the gentrification of urban areas continues, racial residential segregation increases such that lower socioeconomic status populations are forced to live in areas that have substandard conditions both in housing quality and neighborhood characteristics.

Low-income and racial-minority neighborhoods in urban areas often suffer from the highest amount of environmental hazard exposure through air and noise pollution and substandard sanitation (110). This increased prevalence of environmental hazards can contribute to the health impacts of existing energy insecurities. For example, housing with poor ventilation in an area with high levels of air pollution can aggravate a child's asthma. There are well-established, clear disparities in neighborhood rates of asthma due to both indoor and outdoor environmental hazards (50), and there is an association between neighborhoods with energy insecurity and asthma prevalence (22). There is also spatial inequality and disparity in the prevalence of psychosocial stress. Low-income and minority neighborhoods suffer from higher rates of stress, which can compound the negative health effects that result from their already-increased exposure to environmental hazards (e.g., air pollution) (111). For example, family stress combined with exposure to neighborhood violence has been found to increase the incidence of traffic pollution-induced asthma in children, due to the strain on psychosocial pathways (112). The spatial disparities in energy insecurity and health also exist between urban and rural areas, where some low-income rural communities do not have access to natural gas or even electricity services, whereas lack of access to electricity services is rare in urban communities (113).

### Bundled Hardships: Energy and Other Insecurities

As demonstrated by this robust review, energy insecurity is a complex problem, and it does not occur in a vacuum. The hardship of energy insecurity intersects with other hardships, such that each compounds the severity of the others and contributes to detrimental health consequences. Competing needs and hardships, such as food insecurity, water insecurity, and housing insecurity, result in tradeoffs where basic needs are prioritized and sometimes foregone (9, 114, 115). The stress from having to make trade-offs between basic needs for food, water, housing, and energy profoundly affects adult and child mental health (116, 117), which can exacerbate many kinds of physical health and social issues.

With food insecurity, the “heat or eat” dilemma occurs when households must decide whether to expend resources on proper nutrition or adequate energy services because they cannot access or afford both (28, 30, 118, 119). Often, this dilemma leads to undernutrition, especially during the winter and summer months when there are higher energy use needs when it has been found that low-income adults and children have decreased caloric intake compared to lower-energy use months in the spring or fall (28, 118). Other health impacts from food insecurity include acute hospital visits, poor diabetes control, developmental delays, fatigue, and behavioral issues in children (54, 120, 121). In response to high energy bills, people also opt out of medical and dental care, which can lead to worse health outcomes in the future (119).

Water insecurity is another co-occurring hardship. Water and electricity tend to be dependent on one another. On a large scale, hydroelectric dams need water and electricity to function, power plants need cooling water when there are high temperatures, and nuclear plants use large volumes of water (122–124). It is not only water that is needed for energy, but the other way around as well. At the community and household level, access to hot water can be encumbered by energy insecurity (30, 41). When concerned about the cost of energy, some residents may cut back on hot water use (30). Furthermore, water pipes can freeze if there is a fuel shortage or shut-off in time of freezing outdoor temperatures (31). Without water, people's ability to access energy reduces.

Lastly, housing insecurity is a frequently cited competing hardship to energy insecurity (22). The dimensions of housing insecurity include frequent moves, lack of housing options, homelessness, high housing costs, overcrowding, and unstable neighborhoods (125–127). Households that do not have enough money to afford high-quality housing also suffer from an inability to pay high utility bills, which can result in household debt owed to utility companies. Low-income families juggling financial hardships often prioritize other financial obligations such as paying for rent or groceries, seemingly more immediate needs, over paying off debt; this behavior can leave families in prolonged debt cycles (128). Debt owed to utility companies often prevents low-income households from moving because utility debts are not transferable (9), forcing residents to continue living in poor-quality housing. A home cannot be rented without a utility account in the renter's name, which is not possible if they have arrears at another address (9). Frequent moving is also a common form of housing insecurity. Low-income families are five times more likely than higher-income families to experience eviction, resulting in a move (50, 129). Evictions generally occur when rent prices increase beyond what a family is capable of affording. Utility shut-offs play an important role in housing insecurity, as they are often the precursors to eviction. In both instances, households may encounter the double burden of housing *and* energy insecurity (23). As gentrification spreads across US cities, urban housing affordability is unachievable for most low-income families, forcing evictions, moves, overcrowding, and an increase in homelessness, and while newer buildings often enjoy energy-efficiency upgrades, older homes and buildings, which are often less efficient and more expensive to operate from an energy cost perspective, do not receive such upgrades (64, 130). In short, energy cost burdens can increase housing affordability strains whereas lower energy bills can protect against high housing costs and promote residential stability.

## Energy Insecurity, Health, and Coping

Health issues are linked to energy insecurity. In particular, such direct health outcomes are often a result of indoor temperature extremes and inadequate energy access. Thermal stress occurs when residents are unable to heat or cool their homes properly, frequently due to unaffordable utility bills or an inability to access adequate services. As a result of inadequate home energy, residents resort to coping strategies, which, with chronic use, can be taxing and overburden resilience reserves.

### Improvising and Coping Without Energy

Residents implement coping strategies to manage and respond to unmet energy needs; we consider this to be the behavioral dimension of energy insecurity. Despite the ingenuity and agency many people demonstrate in the face of suboptimal energy circumstances, these coping strategies have negative health implications. One such coping strategy is the use of emergency energy technology (i.e., generators), which is generally reserved for disasters but is often employed by energy-insecure households. Generator use is strongly associated with carbon monoxide (CO) fatalities, especially when the generator is placed incorrectly in the household (e.g., in the garage or outside of a bedroom window) (35, 37, 131). Even when placed correctly, generators constantly release CO, which in small, consistent doses can lead to cognitive decline, headaches, nausea, and dizziness. Maintenance issues can aggravate the health impacts of some coping methods, such as poorly maintained households exposing people to higher levels of toxins (50). For instance, some residents living in low-quality housing use unvented gas heaters as their primary heat source and/or hot air units that do not have ducts because they are unable to afford or access improvements. Both practices are associated with increased levels of nitrogen dioxide (NO_2_) and volatile organic compounds (VOCs), which exacerbate allergies and respiratory illness symptoms, create ear, nose, and throat irritation, and contribute to cognitive delays (51, 132). People also resort to avoiding energy sources in their homes or use extreme conservation strategies to reduce energy expenditure. For example, some avoid using a refrigerator, which is associated with undernutrition due to a lack of fresh food in the diet, and/or avoiding hot water use, which can lead to infections and hygiene-related illnesses. Other survival strategies include practices such as only heating one room of the house, going to bed early, and using low lighting (114).

### Cold Stress and Coping

In the winter, cold homes due to a lack of proper heating lead to excess deaths and a number of health problems (13, 47, 69, 99, 100, 133, 134). Cardiovascular symptoms as a result of inadequately heated homes are a prevalent cause of medical issues (69, 135), and rates of hypertension increase in cold temperatures, which can lead to strokes and heart attacks (69, 74, 101, 135). The elderly and people that are already diagnosed with CVD are more at risk of heart attack and stroke due to cold stress (13, 47). Furthermore, research has found that arthritis symptoms worsen in cold homes (12). The rate of pneumonia and other infections, mostly among children, increases due to suppressed immune function from the cold (70), and upper and lower respiratory symptoms, such as coughing and wheezing, are worsened by the cold as well (69, 99, 100). Asthmatic residents and caretakers of asthmatic children living in inadequately heated homes report higher rates of poor well-being and more frequent hospital visits (27, 136). Alzheimer's patients have a higher rate of mortality from a combination of physiological and behavioral factors as a result of the cold (137). Poorer well-being and financial strain from an increased number of medical visits can exacerbate mental health issues, such as depression and anxiety, that may already be heightened due to the stress of energy insecurity (114).

In response to a cold home, many households cope by using alternative heating methods that have a direct impact on health (23). For example, using generators and stoves to provide heat also results in the release of toxic gasses such as NO_2_ and CO that can impair cognitive function, exacerbate respiratory illnesses, and cause mortality (35, 49, 51, 138–140). The use of space heaters or ovens as alternative heat sources can also increase the risk of fire and injury, which could potentially lead to displacement or death (23, 104).

### Heat Stress and Forbearance

Heat stress occurs when households are unable to afford or access energy to cool their homes. The health effects from this type of energy insecurity, such as increased morbidity and mortality rates, are most often seen during heatwaves when excess heat from outside conditions creates heat stress (135, 141, 142). Cardiovascular issues such as heat strokes, hypertension, and heart attack, dehydration, hyperthermia, and nervous system morbidities are examples of health impacts that occur under heat stress (69, 135, 143). Other health effects include a higher likelihood of acute renal failure (42) and increased sleep disturbances as a result of the extreme heat in inadequately cooled homes, which can exacerbate mental health conditions triggered by the stress of energy insecurity (53, 57). This increase in morbidity and mortality is motivated by other social determinants of health that predict energy insecurity (142).

Coping mechanisms for dealing with heat stress have their own related stressors and issues. For example, opening windows for ventilation and relief from heat may seem like an easy, free solution to cool down warm homes; however, in neighborhoods that are perceived to be unsafe, many people cannot or do not travel to cooler locations nor do they leave windows open due to fear of crime and violence (141, 144). Furthermore, open windows expose households to noise pollution, particularly in urban areas where there is high traffic flow, which causes sleep disturbances and obstructed concentration on tasks (57). Open windows also increase the infiltration of outdoor air pollution, such as from motor vehicle exhaust, that is associated with respiratory and cardiovascular health risks (57).

### High-Effort Coping and Resilience Reserves

Energy insecurity plays a role in depleting a person's resilience reserve (24). The *resilience reserve* framework offers a different lens than does past resilience research, which found that marginalized groups were less resilient because they had less social and material support and more life stressors (145). The resilience reserve framework argues instead that marginalized groups that contend with social, economic, medical, physical, and geographic vulnerabilities expend resilience resources to manage everyday hardships, leaving less opportunity to accumulate the psychological and material means with which to respond to and recover from large shocks such as extreme climate events (24). Therefore, after a disaster, marginalized groups have greater difficulties coping and rebounding, because they have already depleted their reserves. For example, years after Hurricane Sandy, which occurred in 2012, low-income NYCHA residents reported longstanding physical and psychosocial difficulties, citing the Hurricane's exacerbation of existing hardships and emotional trauma (24). Specifically, NYCHA residents cited the lack of electricity, heat, and functional elevators as a source of struggle, not just after the Hurricane, but before it as well. Housing, economic, and energy-related hardships had long been a source of chronic stress, constantly gnawing at their resilience reserve before the hurricane hit (24). One risk of an increased frequency of extreme weather events is the potential to exacerbate existing hardships and deeply impact the resilience capacity of vulnerable populations as they confront a growing number of social, economic, health, and energy challenges on a normal basis.

## Discussion

Energy insecurity is a multifaceted phenomenon with short- and long-term iterations influenced by social determinants and a changing climate, ultimately impacting health. This paper reviews existing literature in order to trace the pathways by which chronic and acute energy insecurity directly and indirectly result in various adverse health conditions. Our heuristic model is a unique contribution to the literature that intends to depict seemingly far-flung factors associated with energy, poverty, health, and climate change. We demonstrate the disproportionate effects on vulnerable populations and the mechanisms of household energy that lead to poor health and excess death. Contributors to acute energy insecurity include power outages, fuel shortages, supply issues, and shut-offs stemming from affordability challenges. For the most part, these acute issues are short-lived, though their impact can still be significant for short- and long-term health, well-being, and survival. Meanwhile, the fundamental causes of chronic energy insecurity are rooted in socioeconomic disadvantage as determined by race, income, educational level, position within the life course, and medical conditions that affect energy needs and dependency. It is also deeply affected by housing quality and the concentration of inefficient housing at the neighborhood level that is unfortunately closely patterned along the axes of social inequality and racial residential segregation. The literature suggests that the social determinants of health, housing characteristics, and neighborhood quality seem to predict and/or exacerbate household energy insecurity. As a result, residents turn to coping methods that can have a number of negative health consequences, such as toxic exposure from generators, fires from space heaters, noise pollution and crime from open windows, and many more. Energy production and infrastructure, both globally and locally, contribute to energy insecurity in terms of access and environmental degradation. High energy demand can strain systems, weather events can create power outages, and affordably issues can lead to shut-offs and arrearages. The result of such energy insecurity contributes to outcomes such as psychosocial stress and mental health issues, poor sleep, cardiovascular and respiratory issues, and heat stress, among others. These energy-related difficulties can also deplete people's resilience reserves, such that affected populations are less able to bounce back from acute and chronic hardships. In the context of climate change, more wear-and-tear on the energy systems, housing infrastructure, and population health seems inevitable.

The following discussion offers a critical analysis of the vast but disjointed literature on energy insecurity. One critique of the present literature is that much of it lacks an environmental justice framework, which should be integral to energy insecurity research, and we exemplify this issue by discussing the lack of intersectional consideration of the rising wealth gap, coupled with increasing urbanization, and energy transitions. Second, we explore connections to energy-related issues in the Global South. Although the Global South was not the focus of this review, energy-related issues are prevalent across countries in Africa, Asia, and Latin America and must be taken into consideration when designing interventions, because energy reform anywhere has global implications. Lastly, we discuss the current and future impact of climate change on energy insecurity and the need for greater consideration of climate change when conducting research on energy insecurity. We contend that the use of acute and chronic energy insecurity terminology can be helpful to researchers using a climate change framework because it separates the direct energy-related effects of climate events (acute) from more long-term effects (chronic).

### Wealth Inequality, Urbanization. Energy Transitions, and Environmental Justice

As energy becomes more expensive and the wealth gap increases in the U.S., poorer households may have greater difficulty affording adequate household energy. The difference in the proportion of income allocated to paying for energy bills could grow wider between the rich and the poor; low-income households may increasingly spend a higher proportion of their income on energy bills, because energy bills may increase at a rate faster than does their income (22). In contrast, wealthier households may experience an increase in their income at a rate that can sustain the increased price of energy. Take, for example, the yellow vest protests in France, which were incited by increased fuel costs. Wealth inequality should be addressed in energy insecurity literature, not only to ensure that lower-income households can afford and access energy through evidence-based policy but because socioeconomic status plays a direct role in determining a persons' health environment beyond energy needs.

The growing wealth gap is influenced by the exponential influx of people to urban areas, which do not have adequate infrastructure to provide for the growing population. As urbanization increases, more people are expected to benefit from urban advantage—the idea that there are health benefits to living in urban vs. rural areas (146). However, higher-income urban residents tend to benefit more from the urban advantage, and more often, low-income residents are left in unhealthy, poorly maintained neighborhood and residential environments (146). Thus, poorer residents are left without support and endure intergenerational socioeconomic hardships that prevent families from accumulating wealth. Constantly coping with hardships is financially costly, and high energy bills can be an obstacle to saving money among low-income households (9). Higher-income residents, on the other hand, pay less of their household income toward energy bills and benefit from more efficient and comfortable living environments.

The growing wealth gap between black and white families could also worsen disparate racial impacts as it relates to the intersection of energy, health, and poverty (147, 148). Energy transitions from fossil fuels to renewables such as wind and solar may also contribute to a growing gap because white-collar businesses and wealthier households are able to control and obtain financing for renewable energy, whereas poorer, minority populations are unable to grow their use of renewable energy technology because the cost is prohibitive and access is difficult given the cost, lack of social capital, and lack of education around renewables (130). African Americans have been historically excluded from opportunities for social and economic mobility, and, in the energy sphere, they are also unduly burdened. The literature has failed to explicitly acknowledge the racial divides in energy-related hardship related to cost, comfort, and efficiency and the protracted uptake of the cleanest energy technologies among minoritized groups. It is important to recognize how an increasing wealth gap will perpetuate energy insecurity, further impacting the ability of low-income and minority families to afford adequate energy. Identifying racially based injustices has been critical to advances in environmental justice, and here too, we see a potential for greater analysis of the racial disparities in energy insecurity and related health and social outcomes.

### Energy Insecurity and the Global South

While this review has focused on energy implications in the U.S. and the Global North, many of the same issues are relevant to the Global South. Few articles discussed in this review use a framework of intersectionality to discuss the burden that inordinately affects marginalized populations around the world. Research on global household energy insecurity that uses environmental justice and intersectional frameworks could more adequately analyze this topic. In the Global South, millions of households lack adequate energy sources (149). It is vital that we find methods to expand modern energy services to reach more of the population (150). However, more systematically speaking, energy is dispersed unjustly and inequitably around the world (151, 152). Some populations have greater energy access than they need, while others do not have enough (8). We know that reducing carbon emissions worldwide is vital for addressing climate change, but it is also important to address the unequal distribution of energy sources. Health impacts from energy poverty in the Global South exist partially due to limited access to modern energy technologies. One example is the increased risk of COPD and heart disease from air pollutants that stem from cooking with biomass fuels rather than using electric or gas stoves (7, 19, 153, 154). Households using biomass cookstoves, for instance, face the dilemma of inhaling toxic pollutants from cooking or not eating—both of which have significant health implications. About one-third of the world, almost entirely in the Global South, relies on solid fuel sources such as wood and crop waste for cooking fuel (5, 7). Burning solid fuels for cooking creates indoor air pollution, which is significantly associated with stroke, ischemic heart disease, COPD, lung cancer, and pneumonia. The health impacts of solid-fuel cooking disproportionately impact women and children, who are exposed to higher pollution due to spending a larger amount of their time cooking than do men. Of the 1.3 million COPD deaths among women, about 511,000 are attributable to indoor cooking pollution. In contrast, of the 1.4 million COPD deaths among men, a much smaller proportion−173,000 cases—are attributable to indoor cooking pollution (7). Increasing the prevalence of and access to cleaner fuels for stoves around the world could significantly reduce these negative health outcomes.

In the same way that people of color in the U.S. disproportionately experience energy insecurity, people of color and those living in lower-middle-income countries (LMICs) around the world disproportionately bear the burden of an inequitable global energy system. Globally, people of color bear the burden of household energy insecurity. To this day, 1.3 billion people, most of whom live in Asia, Latin America, and Africa, lack access to modern energy services (155). Of the total number of people lacking electricity access worldwide, 41.3% of the people live in African countries, 28.5% live in India, 27.3% live in other Asian countries, and 2.2% live in Latin American countries (155). Countries of color are also more likely to shoulder the impacts of climate change, though they are less responsible for carbon emissions and environmental degradation, and their ability to withstand and rebuild from weather events is lower than higher-income countries (156).

### Climate Change and Energy Insecurity

The literature reviewed here does not adequately demonstrate a thoughtful link to climate change that goes beyond the concepts of adaptation and mitigation. Future research should examine the impact that climate change will have on energy insecurity. We propose that the concepts of acute and chronic energy insecurity may allow future researchers to expand upon and better evaluate the effects of climate change on household energy. Climate change worsens the direct and indirect health outcomes of energy insecurity and exacerbates cumulative risk, such that those already experiencing energy insecurity are most affected by climate events because they are less able to prepare for, respond to, and recover from disaster events (157). Communities that are most vulnerable to daily hardships are also most vulnerable to the impact of weather events, and the disparity worsens with repeated shocks from climate change (156). For instance, mortality from heatwaves disproportionately affects older, minority, and low-income residents who are less equipped socially, economically, and physiologically to withstand high temperatures. After the 1995 Chicago heatwave, there was clear demographic disparity in mortality rates—lower-income and older people died at much higher rates than the rest of the population (48). These populations were much more vulnerable to heat stress due to living in decaying housing, lack of access to medical services, and social isolation (48). Without movement toward addressing the world's substandard housing, medical, and financial systems, natural disasters could continue to disenfranchise marginalized populations, intensifying and worsening existing stressors. Though some of the literature critically appraised in this review discusses weather events, the vast majority did not explicitly discuss climate change. Research should incorporate and explore the detrimental implications of climate change when evaluating energy insecurity in order to better prepare for future climate scenarios.

While vulnerable populations tend to be hit harder by climate change-related weather events all people are affected by climate change. Climate change-related energy insecurity issues, therefore, could impact anyone regardless of socioeconomic status (158). Severe weather events will lead to acute energy insecurity such as power outages that can affect anyone. More frequent heatwaves will significantly increase energy demand, the need for expanded energy systems, dependence on household air conditioning for entire populations (45, 93, 141, 142, 159). Power outages from heatwaves and storms can put anyone at risk of medical difficulties. Furthermore, storms are increasing in frequency and severity allover the world, putting people at risk of cut off energy access. And regardless of socioeconomic status, people resort to using emergency energy systems (e.g., generators) or non-energy methods during storms or disasters, which puts residents at risk of CO poisoning (160). It is difficult for residents with chronic illnesses to withstand acute energy insecurity from storm-related power outages (141, 161). As shown in these examples, energy insecurity, particularly acute energy insecurity, may become more prevalent for all people as climate change worsens. Notwithstanding the importance of the issue, a demonstrable gap in the literature exists, given that only one-third of the sources included in this review discuss climate change in relation to energy insecurity.

## Strengths and Limitations

This review paper was inspired by a desire to comprehensively understand the predictors and outcomes of energy insecurity. The household energy literature spans many disciplines and research methods. As a result, we drew from a large interdisciplinary pool of research in order to capture enough relevant sources on this topic. The broad inclusion criteria allowed us to find articles that spanned many disciplines and methods to give us a realistic look at the full range of the household energy insecurity literature. Though the breadth of information about household energy is a strength, it was also challenging in that it demonstrated a clear lack of cohesion and systematic guidelines around research on household energy. Therefore, making connections and critiques across these fields of research and sources presented a formidable challenge, though we have done our best to synthesize the literature and draw conclusions from it. The papers vary significantly not only in focus but in scientific quality and rigor—some are more descriptive in nature, while others are more empirical. Many of the studies were not rigorously designed, and for the most part, the literature proved to be quite underdeveloped overall. This review did not assess for quality or eliminate studies on the basis of potential bias. The challenge of a highly dispersed evidence base led us to develop our heuristic model, which attempts to conceptually unify the literature on household energy and health.

## Conclusion

When considering the substantial impact that inadequate household energy can have on population health, we recognize the need to adopt policies and practices that protect people from energy insecurity. This review sought to highlight how energy needs are important for all aspects of daily living and for protection against the effects of acute insecurities in the context of climate change. Climate change threatens life on earth as we know it, and our collective vulnerabilities to energy hardship need to be addressed with extreme urgency (162). By using energy insecurity as a framework for understanding the nexus of effects of unmet household energy needs, we can draw connections between the direct effects of inadequate household energy, such as hypertension from a cold home, and how social vulnerabilities and co-occurring hardships contribute to the problem. With this broader framework, we can begin to understand how policies that address food insecurity, housing insecurity, structural and institutional racism, neighborhood segregation, education inequality, income inequality, and so many other social issues, will also affect energy insecurity and together impact population health. Studying the energy–health–justice nexus through the lens of acute and chronic energy insecurity presents a novel and innovative direction for public health research, advocacy, and policy that can be used to improve the health of people in the U.S. and around the world.

## Author Contributions

SJ, SS, and DH contributed to the conception and design of the review, performed the analysis, contributed to manuscript revision, response to comments from reviewers, read and approved the submitted version of the review, and all accountable of all aspects of the review, including its accuracy and integrity. SJ organized the database and wrote the first draft of the manuscript. SS and DH wrote sections of the manuscript.

### Conflict of Interest

The authors declare that the research was conducted in the absence of any commercial or financial relationships that could be construed as a potential conflict of interest.
